# Improvement of Baro Sensors Matrix for Altitude Estimation

**DOI:** 10.3390/s22187060

**Published:** 2022-09-18

**Authors:** Łukasz Nagi, Jarosław Zygarlicki, Wojciech P. Hunek, Paweł Majewski, Paweł Młotek, Piotr Warmuzek, Piotr Witkowski, Dariusz Zmarzły

**Affiliations:** 1 Faculty of Electrical Engineering, Automatic Control and Informatics, Opole University of Technology, Prószkowska 76 Street, 45-758 Opole, Poland; 2 MovieBird International, 45-667 Opole, Poland

**Keywords:** Kalman filters, EKF, pressure sensors, Barosensors, Tracking Vertical Velocity and Height, Aerial Vehicles, MEA Aircraft

## Abstract

The article presents the use of barometric sensors to precisely determine the altitude of a flying object. The sensors are arranged in a hexahedral spatial arrangement with appropriately spaced air inlets. Thanks to the solution used, the range of measurement uncertainty can be reduced, resulting in a lower probability of error during measurement by improving the accuracy of estimation. The paper also describes the use of pressure sensors in complex Tracking Vertical Velocity and Height systems, integrating different types of sensors to highlight the importance of this single parameter. The solution can find application in computational systems using different types of data in Kalman filters. The impact of pressure measurements in a geometric system with different spatial orientations of sensors is also presented. In order to compensate for local pressure differences, e.g., in the form of side wind gusts, an additional reference sensor was used, making the developed solution relevant for applications such as industrial ones.

## 1. Introduction

Pressure sensors are used in complex systems for Tracking Vertical Velocity and Height [1] in order to calculate velocity, acceleration, angular velocity and angular orientation [2] or to determine the position of various objects in confined spaces [3]. A major problem in complex systems is estimating the uncertainty of the measured data. Typically, in addition to sensors such as GPS, gyroscopes, or accelerometers, an additional, usually single pressure sensor is used [4,5], although the barometric altitude deviates downward from the actual altitude when the flying object is close to the ground. This phenomenon is caused by the ground effect. However, the use of an additional barometric sensor supports the accuracy in determining the object’s position [6]. Pressure itself and its measurement is extremely important for flying objects, both in terms of determining the position and altitude and the effect of the pressure itself on many measured parameters in MEA (More Electric Aircraft) [7] or UAV (Unnamed Aerial Vehicle) [8].

When using many different sensors, there is a need for data synergy. A common solution is the use of Kalman filters. Kalman filters themselves are also used in sensor modeling, for example in estimating the bending angle of a soft sensor [9]. In this article, the authors analyzed a hysteresis loop with multimodal assumptions and realized an accurate observation model with multiple quadratic functions. Other two-stage Kalman filters were used by the authors of the article [10] for a system for a quadrotor with deep neural network processing, where the quadrotor dynamics is considered as a perceptual supplement of the inertial kinematics to improve the precision of multi-sensor fusion. The information encoded in the signal allowed the authors to address issues of IMU error stability, sensor object dynamics and multi-sensor calibration during sensor fusion. In systems where there is high nonlinearity in the modeled system, an Unscented Kalman Filter (UKF) is used to achieve high accuracy and performance [11]. On the other hand, in systems where the Kalman filter introduces diffusion when the model becomes mismatched or noisy, a Multiple Fading Factors Kalman Filter (MFKF) can be used [12]. Another approach to UAV or MEA monitoring is a procedure based on six-position calibration and ellipsoid matching [13]. It is performed to eliminate the bad effects on system accuracy and stability caused by deterministic errors. Data from inertial measurement units (IMUs), global navigation satellite systems (GNSSs), magnetometers and barometers are combined using a sensor fusion algorithm based on the Extended Kalman filter (EKF). The authors’ proposed EKF algorithm is compared with the Unscented Kalman filter (UKF) and unconcentrated quaternion estimator (USQUE) to show its practicality in application.

For more accurate data that is used later, solutions based on several sensors of one type, e.g., pressure sensors [14], in a simple geometric arrangement with basic data-averaging algorithms are also used. With such a solution, the measurement accuracy is much higher. There are also built barometer-IMU systems [15] or systems in which inertial measurement units without gyroscopes (GF-IMUs) or MEMS-IMUs with barometers [16] are used. The latter use accelerometer arrays for angular velocity estimation [17]. On the other hand, the paper [16] describes the use of a Kalman filter for the synchronization of such data. A cascaded, two-stage Kalman filter consisting of separate orientation and position/velocity subsystems was proposed. The performance of altitude tracking is compared with a reference camera-based tracking system.

The quality of the barometric pressure measurement itself is limited by the accuracy of the sensor. Both absolute and relative barometric pressures can vary from device to device due to differences in sensors and their characteristics [18]. However, a properly used barometric sensor allows for precise measurements. The paper [19] describes a highly sensitive sensor for barometric pressure changes. The chosen pressure measurement method is based on microelectromechanical systems’ piezoresistive cantilevers. The high-resolution sensor unit of less than 0.1 Pa in the range of 0.1 to 10 Hz responds to pressure changes with the property of a high-pass filter. For reference, the typical vertical barometric inclination on Earth is 12 Pa/m. A resolution of 0.1 Pa corresponds to a change in altitude of roughly 1 cm.

The paper presents the original novel idea of using multiple pressure sensors arranged in a cube spatial arrangement with appropriately placed air inlets. In addition, computational algorithms were applied to improve the efficiency of the proposed solution. The solution can be applied to systems using computational algorithms that integrate different types of data, for example in Kalman filters [4,20]. The proposed solution allows for the improvement of the estimation accuracy in the barometric altitude estimation module. Applying it to complex Tracking Vertical Velocity and Height systems that integrate different types of sensors will improve the overall precision of parameter estimation in 3D space.

## 2. Materials and Methods

**Figure 1:**
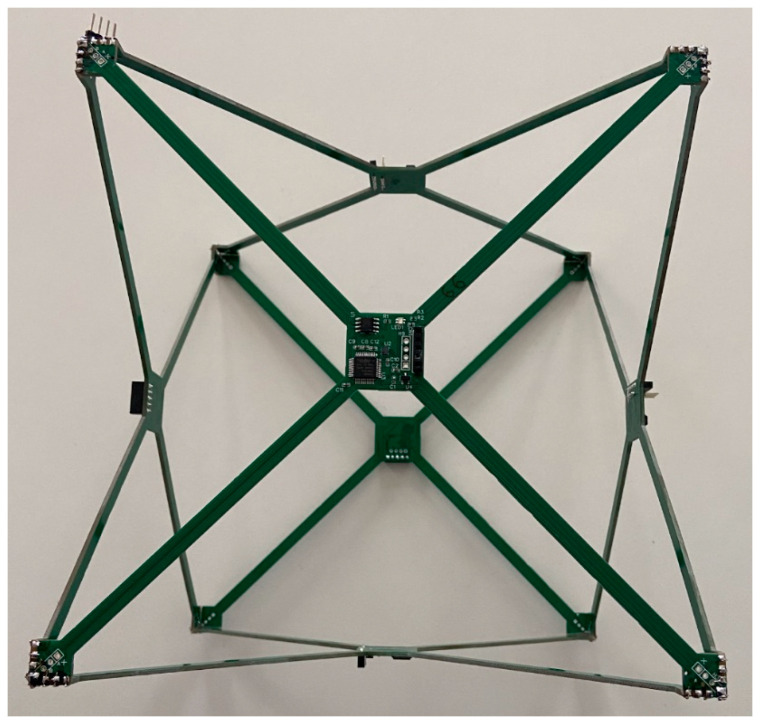
Multipoint pressure measurement system.

**Figure 2:**
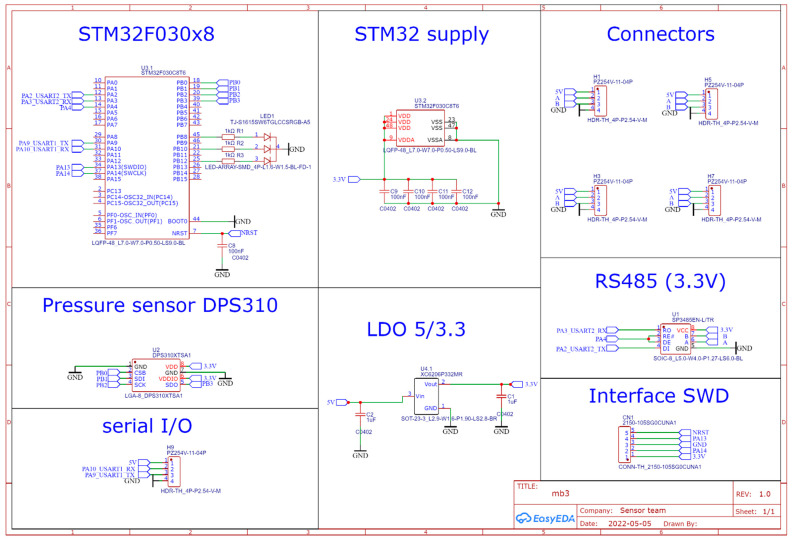
Schematic diagram of the system with the main components of the measurement system (microcontroller and pressure sensor).

A pressure measurement system (Figure 1) was designed and built in a cube arrangement with six Infineon DPS310 sensors mounted on the diagonal cuts of the cube’s side walls. The main components of the sensor system are included in the schematic in Figure 2. The pressure sensors used belong to a group of precision sensors with a high-accuracy class. The parameters of a single sensor are:

- Operation range: Pressure: 300–1200 hPa. Temperature: −40–85 °C.
- Pressure sensor precision: ±0.005 hPa (or ±0.05 m) (high-precision mode).
- Relative accuracy: ±0.06 hPa (or ±0.5 m)
- Absolute accuracy: ±1 hPa (or ±8 m)
- Temperature accuracy: ±0.5 °C.
- Pressure temperature sensitivity: 0.5 Pa/K

**Figure 3:**
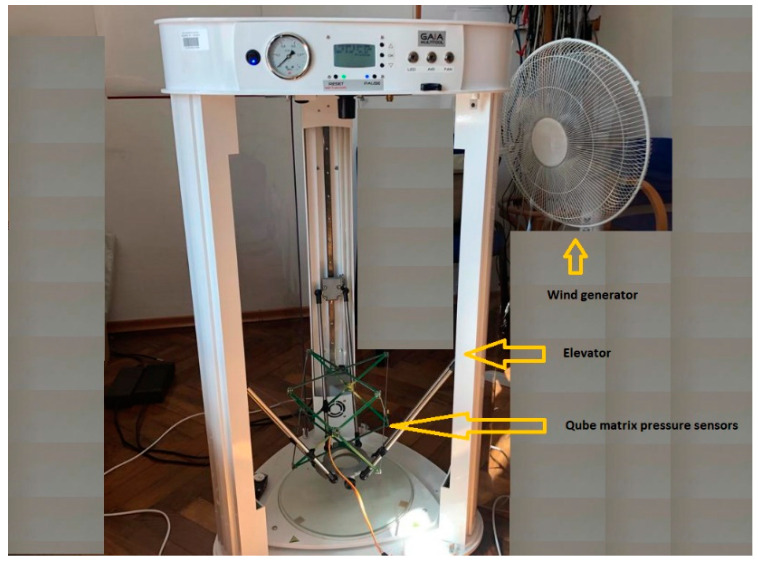
Photograph of the measurement system.

The built system was placed on a moving elevator arm (Figure 3), and the whole system was supplemented with an additional seventh reference pressure sensor, along with a windmill to simulate wind gusts.

**Figure 4:**
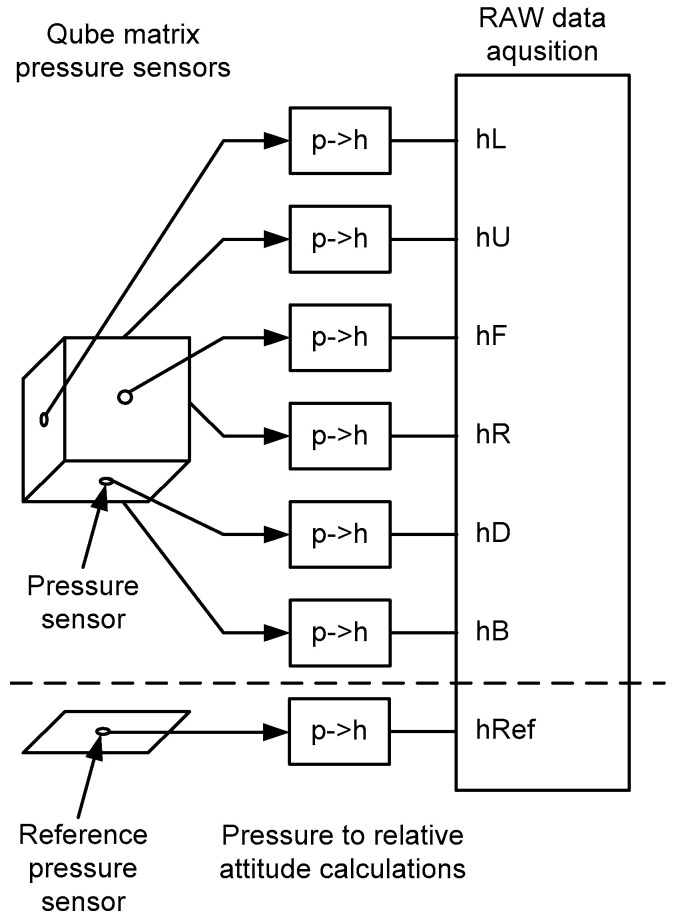
General block diagram of the data acquisition system.

Figure 4 provides a general block diagram of the data acquisition measurement system. Data from the pressure sensors are converted to the relative height, and then a RAW measurement data vector is created, which is subjected to further analysis that is described in the research part of the article.

The conversion of pressure to relative height (block *p*- > h) in the diagram of Figure 4 is implemented according to relation (1):

(1) $$P=P_{b}{\left[\frac{T_{b}+\left(h-h_{b}\right)L_{b}}{T_{b}}\right]}^{\frac{-g_{0}M}{RL_{b}}}$$

where:

$P_{b}$= reference pressure (Pa)

$T_{b}$ = reference temperature (K)

$L_{b}$= temperature lapse rate (K/m) in ISA

h = height at which pressure is calculated (m)

$h_{b}$ = height of reference level b

R = universal gas constant: 8.3144598 J/(mol·K)

$g_{0}$= gravitational acceleration: 9.80665 m/s^2^

M = molar mass of Earth’s air: 0.0289644 kg/mol

**Figure 5:**
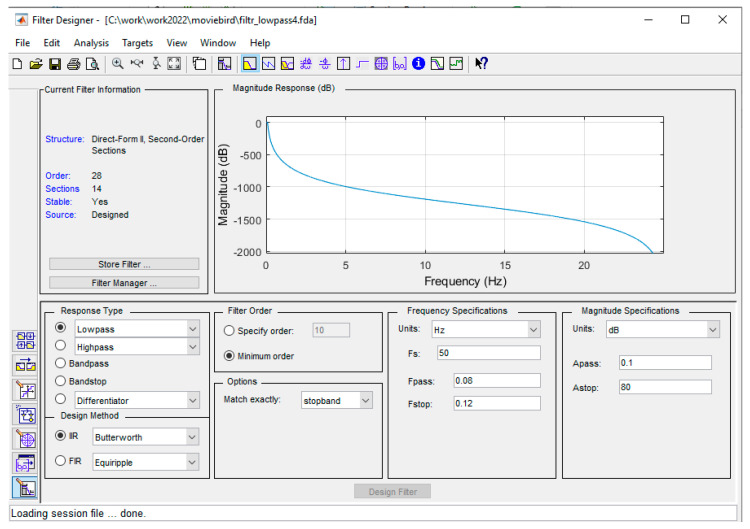
Design of the IIR filter used.

For part of the study, a digital low-pass filter with the parameters shown in Figure 5 was used to process the pressure sensor data.

This filter smooths the waveform of the recorded height over time, as will be shown later in the paper. The filter parameters were chosen experimentally. The filter was designed for a sampling rate of 50 Sa/s. At this rate, the acquisition system provides sensor data. The attenuation in the pass band was minimized to a value of 0.1 dB. The attenuation in the stop band should be as high as possible; −80 dB was set. The filter’s pass band has also been limited to Fstop-Fpass = 0.04 Hz. The frequency response was set at 0.08 Hz, which was a compromise between the effect of smoothing (removing noise) waveforms and the system’s ability to detect changes in pitch over a set period of time. A structure with an infinite impulse response IIR with maximally flat characteristics in the frequency response, i.e., a Butterworth filter, was chosen as the filter. The choice of filter parameters was a compromise between the aim of pursuing the assumptions described above and the increase in the size of the filter, which increased the computational effort and could cause instability in the filter response. The filter used is only an example to illustrate the desirability of its use. This filter should be redesigned for a specific implementation solution depending on the design assumptions of a given system, e.g., the system response time, set level of de-noising, computational capability of the system, etc.

## 3. Results

**Figure 6:**
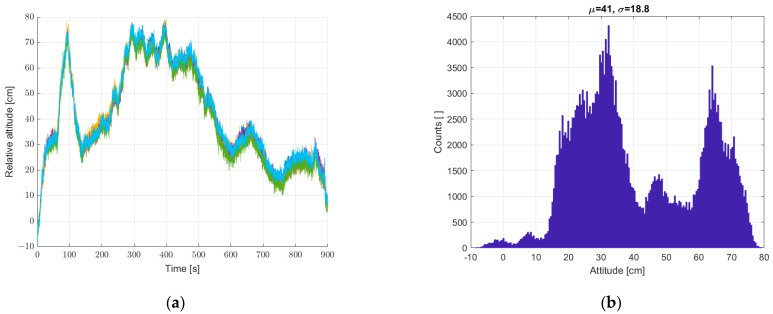
(**a**) Graph over time from six sensors without a reference sensor, and (**b**) histogram created from a single data vector merged from data vectors from six sensors. Static measurements—sensor array at standstill.

Experimental tests were carried out in the measurement system of Figure 3, adopting the following scheme of operations:

The scatter of relative height measurements, based on pressure measurements from six sensors placed in the cube arrangement described in the earlier chapter (Figure 1), was checked. The results are shown in Figure 6. The set of sensors remained static. Significant height variations were recorded due to small pressure fluctuations caused by local disturbances (ground effect).

**Figure 7:**
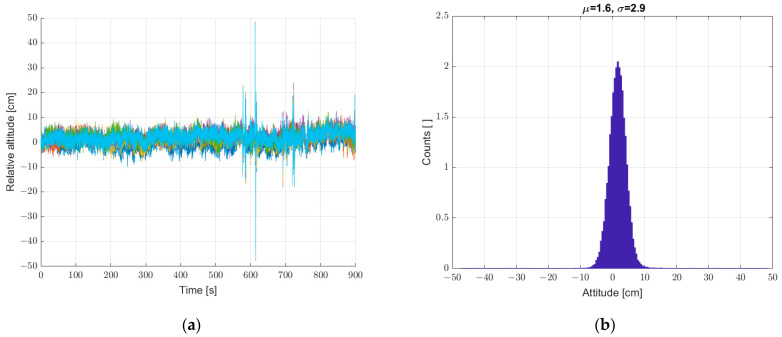
(**a**) Graph over time from six sensors with correction from reference sensor, and (**b**) histogram. Static measurements—sensor array at standstill.

On this basis, we decided to implement a reference sensor. All subsequent measurements were made with reference to the static seventh reference sensor. The relative height registrations of the six sensors in all subsequent measurements are calculated as the difference of the determined height from a given sensor (from 1 to 6) and the seventh sensor. Next, a static measurement (a set of sensors at standstill) was performed with the reference sensor included, according to Section 2. The results are shown in Figure 7. The implementation of the reference sensor significantly reduced the scatter of determined heights for static measurements. There is still a “noise” effect and “impulse” interference.

**Figure 8:**
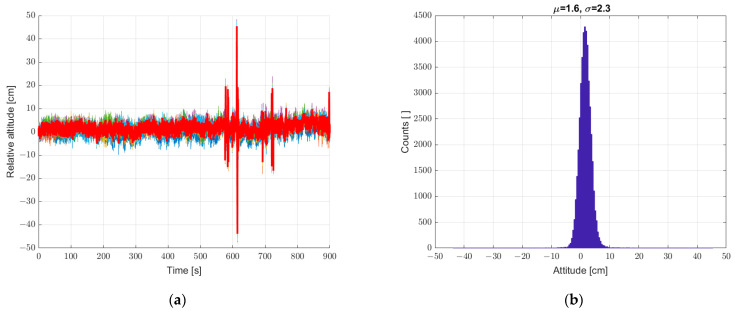
(**a**) Graph over time from 6 + 1 sensors, graph with a bold line calculated from the average of the individual sensors, and (**b**) histogram. Static measurements—sensor array at standstill.

The next step was to verify the possibility of reducing the described interference by averaging the measurements from six sensors geometrically arranged in a way that leveled the spatially inhomogeneous pressure fluctuations and the wind gusts caused by this phenomenon. The results of these tests are shown in Figure 8. Averaging the sensor data reduced the scatter of the height measurement; the recorded standard deviation decreased from a value of 2.9 to 2.3. In the conducted experiment, forced air motion was not yet implemented. The recorded fluctuations came from small pressure fluctuations recorded in the closed room in which the measurement system was placed.

The proposed system neutralizes impulse noise and interference as well as low-frequency fluctuations in the instantaneous value of the measured height in two ways:

- in the hardware part:

Through a geometric arrangement of sensors in a cubic cube (six sensors) with an additional seventh (+1) reference sensor, as shown in the block diagram of Figure 4.

- in the software part:

By correcting the instantaneous fluctuations of the determined heights from the six cube sensors based on the measurements from the seventh reference sensor (Figure 4):

(2) $$\left[\begin{array}{c} \begin{array}{c} h^{\prime }{}_{L}\\ h^{\prime }{}_{U} \end{array}\\ \begin{array}{c} h^{\prime }{}_{F}\\ h^{\prime }{}_{R} \end{array}\\ \begin{array}{c} h^{\prime }{}_{D}\\ h^{\prime }{}_{B} \end{array} \end{array}\right]=\left[\begin{array}{c} \begin{array}{c} h_{L}\\ h_{U} \end{array}\\ \begin{array}{c} h_{F}\\ h_{R} \end{array}\\ \begin{array}{c} h_{D}\\ h_{B} \end{array} \end{array}\right]-h_{Ref}$$

where: *h_L_*_…*B*_—heights determined from pressure sensors placed on the walls of the cube, *h_Ref_*—the height determined for the reference sensor, and *h*’*_L_*_…*B*_—corrected heights relative to the height determined from the reference sensor *h_Ref_*.

By averaging (corrected according to the above description—Formula (2)) the measurement data from six sensors on a cubic cube:

(3) $$\bar{H}=\frac{1}{6}\left(h^{\prime }{}_{L}+h^{\prime }{}_{U}+h^{\prime }{}_{F}+h^{\prime }{}_{R}+h^{\prime }{}_{D}+h^{\prime }{}_{B}\right)$$

where: $\bar{H}$—average value of adjusted heights.

And by digital filtering with the designed filter (with amplitude characteristics shown in Figure 5 (Design of the IIR filter used)):

(4) $$H_{F}\left[n\right]=\frac{1}{a_{0}}\left(\overset{P}{\underset{i=0}{\sum }}b_{i}\bar{H}\;\left[n-i\right]+\overset{Q}{\underset{j=1}{\sum }}a_{j}H_{F}\;\left[n-j\right]\right)$$

where: *P* is the feedforward filter order, *b_i_* are the feedforward filter coefficients, *Q* is the feedback filter order, *a_j_* are the feedback filter coefficients, $\bar{H}$ [*n*] is the *n*-th sample in discrete time of $\bar{H}$ introduced in (3), and *H_F_* [*n*] is the *n*-th sample in discrete time of the filtered altitude.

**Figure 9:**
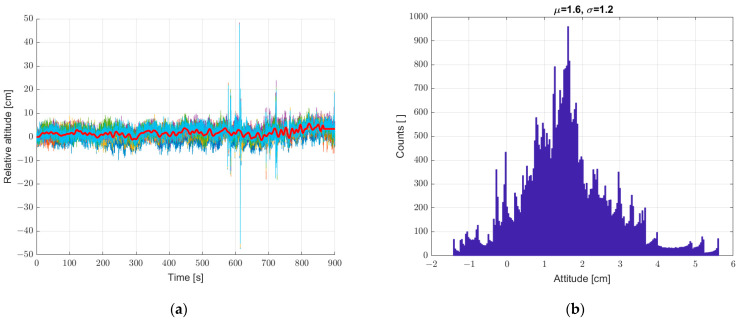
(**a**) Graph over time with 6 + 1, bold graph calculated from data averaged and filtered with a low-pass IIR filter, and (**b**) histogram. Static measurements—sensor array at standstill.

The averaging of sensor data reduced the scatter of height measurements, and the next step was to implement an additional method for processing the recorded data. In order to further smooth the waveform of recorded data and minimize the standard deviation parameter of static measurements, we decided to use digital filtering with the filter described in the earlier chapter. The results of the analysis are shown in Figure 9. The use of digital filtering results in a further favorable reduction of the standard deviation from 2.3 to 1.2, probably at the expense of the dynamics of recording the height of the system in motion, which will be checked in later analyses.

**Figure 10:**
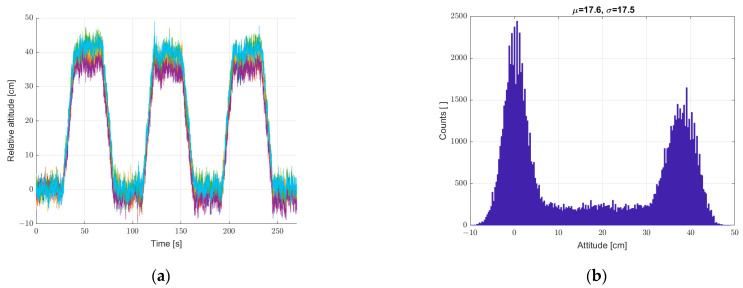
Graph over time from (**a**) 6 + 1 sensors, and (**b**) histogram. Dynamic measurements—sensor array in motion.

**Figure 11:**
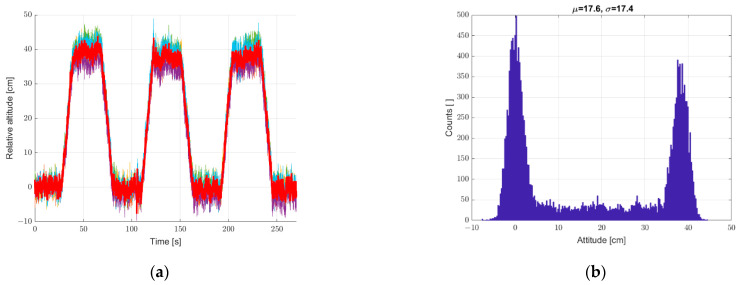
(**a**) Graph over time from 6 + 1 sensors, a bold graph calculated from the average of individual sensors, and (**b**) histogram. Dynamic measurements—sensor array in motion.

**Figure 12:**
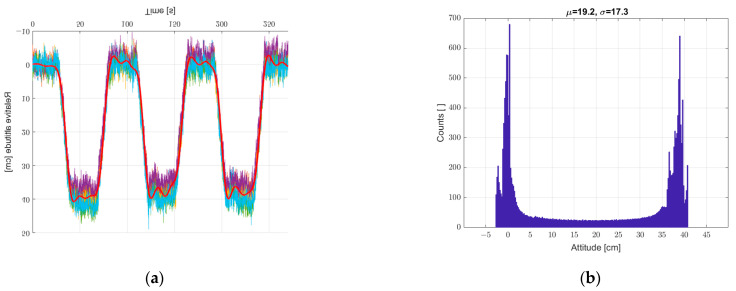
(**a**) Graph over time with 6 + 1, bold graph calculated from data averaged and filtered with IIR low-pass filter, and (**b**) histogram. Dynamic measurements—sensor array in motion.

Another study was carried out for the system put into motion caused by the periodic movement of the elevator arm from Figure 3 in the vertical direction in the range of 0–400 mm relative to the static seventh reference sensor. The results are shown in Figure 10, Figure 11 and Figure 12, in a system analogous to the static tests of Figure 7, Figure 8 and Figure 9. Additionally, for dynamic measurements, an improvement in the accuracy of the height estimation was noted in successive data processing steps (averaging, filtering). The histograms additionally show two maxima representing two height points where the elevator with six pressure sensors was parked (stopped for a long moment).

The tests conducted until now were carried out in a closed room without forced air movement. The next two sets of tests present results for forced air movement with the windmill from Figure 3. The windmill was intended to simulate difficult realistic measurement conditions with potential applications of the system in an open space. The windmill periodically oscillated the blowing direction +/−30 degrees. The speed of the generated wind was set at 3.0 m/s.

**Figure 13:**
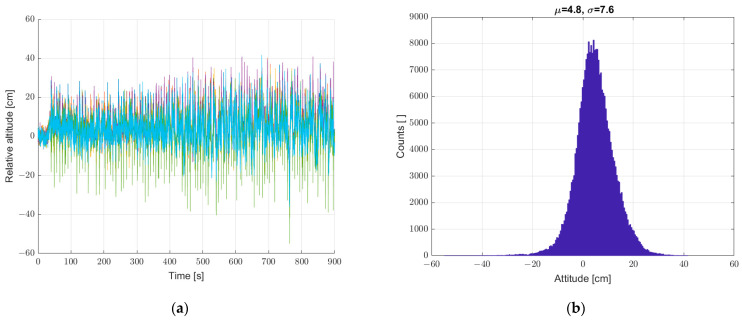
(**a**) Graph over time from 6 + 1 sensors, and (**b**) histogram. Static measurements—sensor array at standstill, with additional variable crosswind gusts.

**Figure 14:**
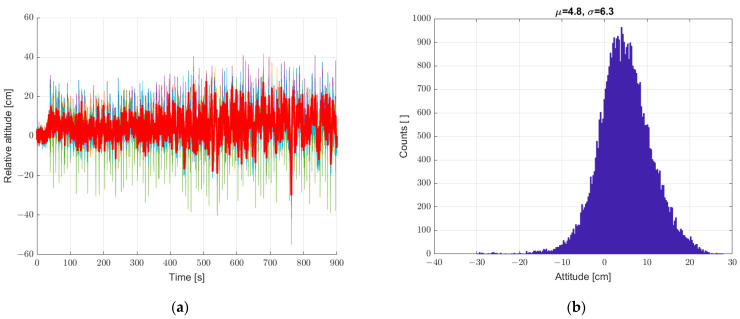
(**a**) Graph over time from 6 + 1 sensors, a bold graph calculated from the average of the individual sensors, and (**b**) histogram. Static measurements—sensor array at standstill, with additional variable crosswind gusts.

**Figure 15:**
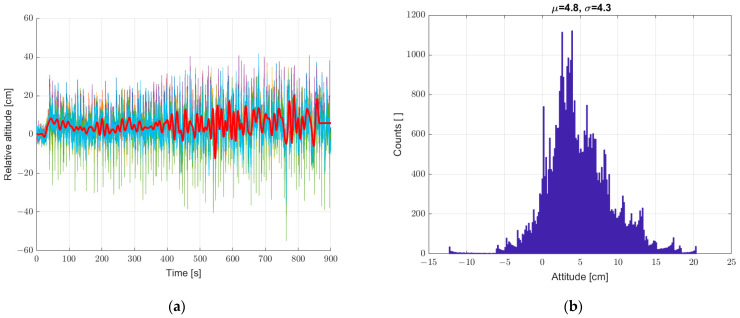
(**a**) Bold graph calculated from data averaged and filtered with an IIR low-pass filter, plotted over time with 6 + 1, and (**b**) histogram. Static measurements—sensor array at standstill, with additional variable crosswind gusts.

The first set of measurements were static tests (sensor system at standstill), as shown in Figure 13, Figure 14 and Figure 15.

**Figure 16:**
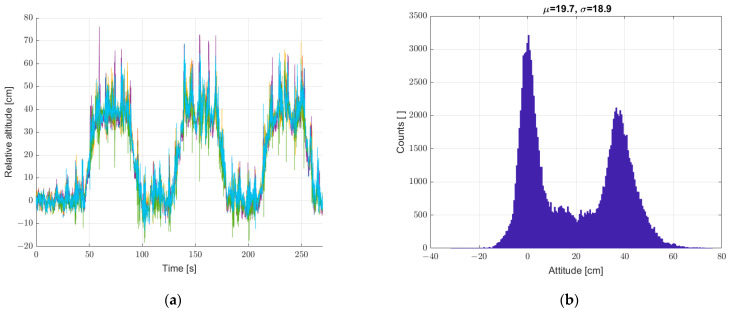
(**a**) Graph over time from 6 + 1 sensors, and (**b**) histogram. Dynamic measurements—sensor array in motion, with additional variable crosswind gusts.

**Figure 17:**
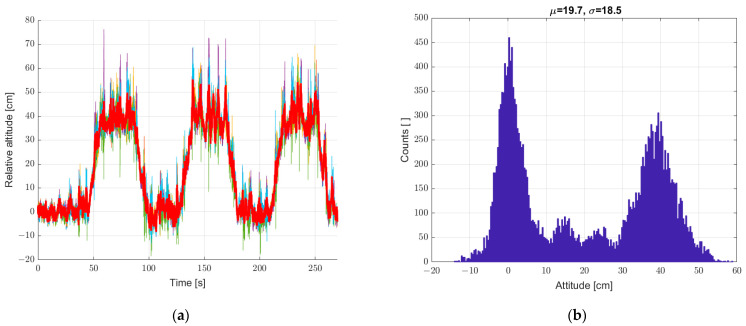
(**a**) Graph over time from 6 + 1 sensors, bold graph calculated from the average of individual sensors, and (**b**) histogram. Dynamic measurements—sensor array in motion, with additional variable crosswind gusts.

**Figure 18:**
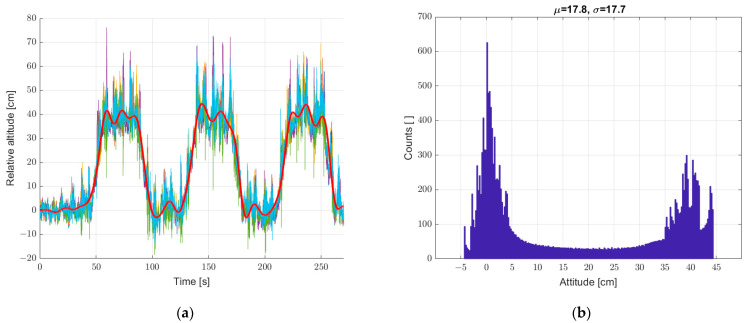
(**a**) Graph over time with 6 + 1 bold plot calculated from data averaged and filtered with IIR low-pass filter, and (**b**) histogram. Dynamic measurements—sensor array in motion, with additional variable crosswind gusts.

The second set of measurements are dynamic tests (sensors in motion), as shown in Figure 16, Figure 17 and Figure 18.

## 4. Discussion

Based on the study, the following conclusions were formulated:

- in precision positioning systems, the use of pressure sensors without reference pressure measurements, where the required precision of the height estimation is of the order of single centimeters, seems to be unjustified,
- in precision positioning systems using Kalman methods, which integrate measurements from various types of sensors, e.g., pressure, acceleration, magnetometers, the presence of reference pressure sensors will contribute to the system to improve height estimations,
- the use of additional reference pressure sensors with a known location allows one to significantly reduce the impact of momentary local pressure fluctuations, and as a result, height measurements can be carried out with an accuracy of the order of single centimeters,
- the use of pressure measurements with reference sensors in complex multi-sensor systems, in which pressure-based height measurements are one of several integrated methods of height measurement, will allow one to increase the precision of height estimations (The analysis and evaluation of precision improvement in such systems will be the subject of further research by the authors.),
- the use of an array of pressure measurement sensors in a geometric arrangement with different spatial orientations of the sensors (The paper proposes the author’s arrangement of a cube with sensors placed at the intersection of the diagonal squares of each side of the cube) compensates for local pressure differences in the form of crosswind gusts, which is important for practical industrial applications of the system,
- in the presented study, despite the use of a reference sensor, fast-variable height fluctuations (noise) and a long-term trend (an apparent shift in the average value with a static sensor array) are still observed, as well as a constant height estimation error of several centimeters,
- the fast-varying height fluctuations (noise) are probably caused by several phenomena: measurement noise of sensors, local fast-varying waves of pressure fluctuations in the infrasound region in combination with delays in pressure registration in moving and reference sensors (pressure measurements take place sequentially),
- the observed long-term trend observed in the form of a shift in the average value with static measurements is probably caused by the measurement instability of the sensors themselves, which in turn may be largely due to temperature fluctuations of the individual sensor system,
- the observed constant height estimation error of several centimeters is probably caused by the nonlinearity of the processing of pressure measurement sensors. The sensor DPS310 is a calibrated sensor and contains seven calibration coefficients. These were used in the application to compensate for sensor nonlinearities in the measurement results. The absolute pressure accuracy from the sensor specification is +/−6 Pa in the range of Ta = 20 + 60 °C.

Further improvements in the estimation accuracy can probably be achieved by:

- minimizing, if possible, the distance between the pressure sensors, which are placed on the moving object and the reference sensors. We think that the selection of optimal distances between sensors can be the subject of future studies,
- stabilizing the thermal operation of individual sensors (results are subject to thermal drift of sensors),
- increasing the number of pressure measurement sensors on the moving object and reference sensors,
- the use of differentiation in the spatial orientation of sensors in addition to that already used,
- software linearization through physical multi-point height measurements for each individual sensor used,
- use of sensors with the highest possible precision and stability of measurements, assuming a given budget for the sensor part of the system under construction.

The authors, in future works, will attempt to carry out further optimizations, as described above.

## Data Availability

The study did not report any data.
